# Air Pollution, Aeroallergens, and Emergency Room Visits for Acute Respiratory Diseases and Gastroenteric Disorders among Young Children in Six Italian Cities

**DOI:** 10.1289/ehp.0900599

**Published:** 2009-08-13

**Authors:** Flavia Orazzo, Luigi Nespoli, Kazuhiko Ito, Davide Tassinari, Daniela Giardina, Maurizio Funis, Alessandra Cecchi, Chiara Trapani, Gisella Forgeschi, Massimo Vignini, Luana Nosetti, Sabrina Pigna, Antonella Zanobetti

**Affiliations:** 1 Pediatric Emergency Room, Santobono’s Hospital, Naples, Italy; 2 Pediatric Emergency Room, Pediatric Department, University of Varese, Varese, Italy; 3 Nelson Institute of Environmental Medicine, New York University School of Medicine, New York, New York, USA; 4 Pediatric Emergency Room, Pediatric Department, University of Bologna, Bologna, Italy; 5 Pediatric Emergency Room, Maggiore’s Hospital, Bologna, Italy; 6 Pediatric Emergency Room, Pediatric Department, Torre Galli, Florence, Italy; 7 Pediatric Emergency Room, Mayer Hospital, Florence, Italy; 8 Pediatric Emergency Room, Ponte a Niccheri Hospital, Florence, Italy; 9 Pediatric Emergency Room, Pediatric Department, Salesi Hospital, Ancona, Italy; 10 Pediatric Emergency Room, Gallarate Pediatric Hospital, Gallarate, Italy; 11 Department of Environmental Health, Harvard School of Public Health, Boston, Massachusetts, USA

**Keywords:** air pollution, asthma in children, epidemiology of asthma, children’s health

## Abstract

**Background:**

Past studies reported evidence of associations between air pollution and respiratory symptoms and morbidity for children. Few studies examined associations between air pollution and emergency room (ER) visits for wheezing, and even fewer for gastroenteric illness. We conducted a multicity analysis of the relationship between air pollution and ER visits for wheezing and gastroenteric disorder in children 0–2 years of age.

**Methods:**

We obtained ER visit records for wheezing and gastroenteric disorder from six Italian cities. A city-specific case–crossover analysis was applied to estimate effects of particulate matter (PM), nitrogen dioxide, sulfur dioxide, ozone, and carbon monoxide, adjusting for immediate and delayed effects of temperature. Lagged effects of air pollutants up to 6 prior days were examined. The city-specific results were combined using a random-effect meta-analysis.

**Results:**

CO and SO_2_ were most strongly associated with wheezing, with a 2.7% increase [95% confidence interval (CI), 0.5–4.9] for a 1.04-μg/m^3^ increase in 7-day average CO and a 3.4% (95% CI, 1.5–5.3) increase for an 8.0-μg/m^3^ increase in SO_2_. Positive associations were also found for PM with aerodynamic diameter ≤ 10 μg and NO_2_. We found a significant association between the 3-day moving average CO and gastroenteric disorders [3.8% increase (95% CI, 1.0–6.8)]. When data were stratified by season, the associations were stronger in summer for wheezing and in winter for gastroenteric disorders.

**Conclusion:**

Air pollution is associated with triggering of wheezing and gastroenteric disorders in children 0–2 years of age; more work is needed to understand the mechanisms to help prevent wheezing in children.

Mounting evidence indicates that air pollution plays an important role on morbidity and mortality in all ages and especially in children. Many studies have focused on the association between pollutants and adverse respiratory health effects in children around the world (Bates 1995; Bedeschi et al. 2007; Dockery et al. 1996; Loomis et al. 1999; Ostro et al. 1999; Romieu et al. 2002; Thurston et al. 1997; Vigotti et al. 2007). In a European review, Valent et al. (2004) reported that among children 0–4 years of age, between 1.8% and 6.4% deaths could be explained by outdoor air pollution, whereas acute lower respiratory tract infections due to indoor air pollution accounted for 4.6% of all deaths and 3.1% of disability-adjusted life-years (DALYs). Recently, epidemiologic studies have also suggested that the effects of air pollution, at current levels, are particularly pronounced in the first years of life (Brauer et al. 2002).

Children are especially susceptible and may be more exposed than adults to ambient air pollution, partly because children have higher ventilation rates than adults and because they tend to spend more time outdoors. Gastroenteritis is an inflammation of the gastrointestinal tract. The inflammation can be caused by infection with certain viruses, bacteria, or toxicants or by adverse reaction to ingested material or medication. Inhaled environmental pollutants in the first ages of life can have profound impacts on the interrelationships between signaling molecules and their targets, thereby upsetting homeostasis in the lung and possibly in the intestine (Kasper et al. 2005).

A few multicity studies have investigated the short-term effects of air pollutants on the development of respiratory infections and wheezing in very young children, using a case–crossover analysis or time-series analysis (Barnett et al. 2005; Bedeschi et al. 2007; Galan et al. 2003; Lin et al. 2003; Luginaah et al. 2005; Romeo et al. 2006; Tobias et al. 2003; Vigotti et al. 2007). However, none has studied gastroenteric diseases, which represent a major fraction of morbidity outcomes in children, including visits to the emergency room (ER).

Air pollution is a concern in Italy, and several studies of mortality and hospital admissions in adults (Katsouyanni et al. 1996) and children (Bedeschi et al. 2007; Romeo et al. 2006; Vigotti et al. 2007) have addressed this issue.

In this study, we examined the association between air pollution and pediatric hospital ER visits for wheeze and gastroenteric disorders among children 0–2 years of age in six Italian cities between 1996 and 2002.

We applied a multicity case–crossover analysis to study the acute effect of particulate matter with aerodynamic diameter ≤ 10 μg (PM_10_), nitrogen dioxide, sulfur dioxide, ozone, and carbon monoxide, and aeroallergens (Graminaceae and Urticaceae) on the risk of ER visits for wheezing and gastroenteric disorders among children 0–2 years of age, and we examined whether that risk was modified by season.

## Data and Methods

### Health data

We examined the association between air pollution and daily pediatric hospital ER visits of children 0–2 years of age living in six Italian cities: Ancona (west on the sea), Bologna (center), Padua (north), Varese and Gallarate (north), Florence (center), and Naples (south). Varese and Gallarate were analyzed as one because these are two small municipalities near to each other in a zone with several industries in the north of Italy.

We collected information on daily ER visits for wheezing (Castro-Rodriguez et al. 2000) for the years 1996–2000 from the main pediatric hospitals in each city. Pediatric doctors in the ER collected information through questionnaires administered to the family when they were bringing their children to the ER.

We extracted daily counts of wheezing, defined as respiratory disease of lower airways (Martinez 2005). Wheezing resembles a musical sound generated by the high-speed airflow through the lumen that obstructs the airways. The children present rhinitis with coughing, and dyspnea; the chest is enlarged. Soft rantoles and wheezing, especially at the end of inspiration, are detected through auscultation.

We also extracted gastroenteric disorders, defined as acute enteric disease with diarrhea and vomiting (Elliott and Dalby-Payne 2004). In the study, children were excluded if they accidentally ingested poisonous substances, had urinary infection, or had gastroesophageal reflux.

### Environmental data

Air pollution data were obtained from the Italian Environmental Protection Agency ARPA (Agenzia Regionale per la Protezione Ambientale) for the six cities during the years 1996–2002.

We analyzed ambient PM_10_ (available in Florence, Bologna, and Naples), total suspended particulates (TSP) (available in Ancona, Varese, Padua), NO_2_, SO_2_, O_3_, and CO.

PM_10_ and TSPs were measured by β attenuation, SO_2_ by pulse fluorescence, NO_2_ by chemiluminescence, O_3_ by ultraviolet absorption, and CO by infrared absorption. Pollutants concentrations were expressed as 24-hr means for TSP, PM_10_, SO_2_, and NO_2_ and as the maximum of 8-hr means between 0800 and 1600 hours for CO and O_3_. The 24-hr and 8-hr averages were computed if at least 77% of the measures were available for all the pollutants.

We transformed the TSP data in PM_10_ in those cities where only TSP was available using the conversion factor (PM_10_ = 0.83 × TSP) suggested by the 1999 Council Directive of European Commission (Council Directive EC 1999).

Many of the cities have more than one monitoring location, and we computed local daily mean pollution concentrations as the average of all monitors in the city. We obtained local mean temperature and relative humidity from the same monitoring stations that collected air pollution data. We also obtained data on aeroallergens such as Gramineae and Urticaceae. The levels of these airborne pollens were collected using a volumetric spore trap (VPPS 2000; Lanzoni Co., Bologna, Italy) located on the rooftop of each city’s central station. Daily pollen counts were converted into 24-hr average concentrations expressed as grains per cubic meter. During the study period, daily pollen data were available from April to September in each city, and in Naples, Ancona, and Varese-Gallarate for all year. Because pollen data were very sparse during winter, this analysis was performed only in the summer.

### Statistical methods

We investigated the association between daily air pollution concentrations and emergency visits for wheezing and acute enteric disease in children using a case–crossover design (Maclure 1991). The case–crossover design samples only case days, and a case subject becomes a control subject on days without event, in this analysis ER admission. By using control days close in time to the event day, there is no confounding by slowly varying personal characteristics, because each subject is the perfect match for himself. Moreover, the case–crossover method controls for long-term trend and seasonality by design. Air pollution has short-term serial correlation, and to ensure that all of our control days were independent, we chose control days matched on day of the week in the same month and year as the event day. In addition, for a sensitivity analysis, we conducted case–crossover analysis by matching on every third day from the case day in the same month and year, which provides more control days. In the every-third-day referent sampling method, day-of-week variable was included in the regression model.

To control for potential impacts of weather, we used same-day mean temperature to control for immediate effects and the average of the lags 1–3 of mean temperature to represent the delayed effects. Because risk may vary nonlinearly with temperature, we used natural cubic spline (with three degrees of freedom) for both the same day and the moving average of the previous 3 days. Both temperature terms (same day and lag 1–3) were included simultaneously in the models. We also included a natural cubic spline with three degrees of freedom to control for relative humidity. Because the relationship between air pollution and wheeze or gastroenteric illness may change across seasons, we also conducted stratified analyses by season, defined as summer for the months of April–September and winter as October–March. Air pollution was modeled linearly. We analyzed the effect from the same day up to 6 prior days; we also computed the moving averages as averages of the exposure lags. For example, the 2-day moving average (lag 0–1) was computed as the mean of the same and previous days; the 3-day moving average (lag 0–2) included lag 0, 1, and 2, and so on, up to the 7-day moving average (lag 0–6), which is the average of lag 0–6 days.

The analysis was conducted in each city separately. To estimate an average effect for all cities, the city-specific results were combined using a random-effect meta-analysis using the method of DerSimonian and Laird (1986). We also report the *p*-values for the test of homogeneity. The results are expressed as percentage increase in each outcome for an interquartile range (IQR) increase in exposure. The IQRs were computed as the average IQR across the cities. The data were analyzed using a conditional logistic regression (PROC PHREG release 8.2; SAS Institute Inc., Cary, NC, USA).

## Results

The six cities analyzed in this study span north, central, western, and southern regions of Italy and present differences in terms of weather and population. The largest among the six cities is Naples, with a population of around 1 million. The smallest city, Gallarate (population ~ 50,000), and the second smallest city, Varese (~ 90,000), were combined for the analysis. Thus, Ancona (population ~ 100,000) effectively had the smallest population in our analysis. The population of children 0–2 years of age was about 2% of the total population in these cities.

Table 1 shows the daily mean and SD of ER visits for wheeze and gastroenteric illness for all year and by season. The mean number of emergency visits for total wheezing varies between 18 in Naples, the largest city, and < 1 in Ancona, the smallest city. The daily counts for wheeze are generally larger in cold season than in warm season, whereas for gastroenteric illness, there is little seasonal pattern.

Table 2 shows the distribution of the weather variables and air pollution. The weather is relatively mild, with the mean temperature ranging from 12.7°C in Varese–Gallarate (north) to 18.6°C in Naples (south). The mean levels of gaseous air pollutants varied by a factor of two across these six cities, with Naples showing the highest levels, whereas the mean levels of PM were less variable. The number of missing values varies by city, with Varese and Gallarate being the city with the highest percentage of missing values (between 1% and 22%). Across the other cities, the percentage of missing values varied between 0 and 8%.

Associations between air pollutants and ER visits for both wheezing and gastroenteric disorders were positive at all single-day lags (result not shown) but consistently less significant than those for moving averages. (For SO_2_ only we found significant associations from lag 2 to lag 6.) Therefore, we present the result using moving averages.

Table 3 shows the combined results for total wheezing for all the moving averages. Among the air pollutants, CO was most strongly associated with ER visits for wheezing, followed by SO_2_. However, generally positive associations were found for PM_10_ and NO_2_ as well, and, although some associations were not statistically significant, for all the pollutants considered the estimated risks increased as the average of longer lags were considered. For CO, the estimated risks were significant for all the moving averages analyzed. For example, the percentage excess risk estimate for the lag 0–6 (i.e., the average of 0- through 6-day lags) was 2.7% [95% confidence interval (CI), 0.5–4.9] in total wheezing for a 1.04-μg/m^3^ increase in the average of 0- through 6-day lags of CO. The strongest association between ER visits for wheezing and SO_2_ was found for the lag 0–7, with a 3.4% (95% CI, 1.5–5.3) increase for a 8.0-μg/m^3^ increase in SO_2_. No significant associations were found with O_3_.

The associations between air pollution and ER visits for gastroenteric disorders (Table 4) were generally weaker than those for wheezing. CO and SO_2_ showed significant associations, but unlike the result for wheezing, the estimated risks for CO were not consistently larger for the moving averages with longer lags. The strongest association for CO was found for the 3-day moving average (i.e., average of 0- through 2-day lags, lag 0–2), with a 3.8% increase (95% CI, 1.0–6.8) per 1.1 μg/m^3^ increase in CO. For SO_2,_ NO_2_, and PM_10_, the estimated risks were larger for the moving averages with longer lags, although significant associations were found only for the lag 0–6 and lag 0–7 of SO_2_. No significant associations were found with O_3_.

Tables 3 and 4 also present the *p*-values for homogeneity; although for total wheeze we found significant (at significance level of 0.05) heterogeneity in PM_10_ and NO_2_, not much heterogeneity between the cities was found for gastroenteric disorders.

The results from the sensitivity analyses in which control days were chosen from every third day from the case day in the same month and year show the pattern of associations (the lag structure and relative strength of associations across pollutants) similar to that of the main analysis, but the strength of associations is somewhat weaker in the sensitivity analysis despite larger number control days.

**Figure 1:**
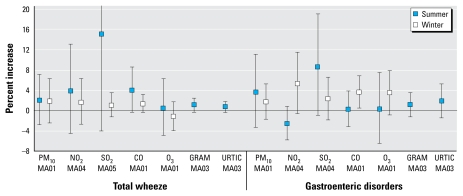
Combined results by season for total wheeze and gastroenteric disorders. Abbreviations: GRAM, Graminaceae; URTIC, Urticaceae. Results are expressed as percent increase (95% CI) in risk of wheeze and gastroenteric disorders for an IQR increase in air pollution. The results by season for selected moving averages (MA) are presented in the *x*-axes for each outcome (total wheeze and gastroenteric disorders) and for each pollutant for lag days 1, 3, 4, and 5.

When data were stratified by season (Figure 1), for wheezing, the risk estimates for NO_2_, SO_2_, and CO were larger in summer than in winter. However, the CIs for these estimates were wide, and therefore these contrasts were not statistically significant. For gastroenteric disorders, the estimated risks for NO_2_ and CO were larger in winter than in summer, although, again, these differences were not statistically significant.

The results for aeroallergens during summer are reported in Tables 3 and 4 and in Figure 1. Unlike air pollutants, the extent of lagged associations between the pollen and ER visits were shorter, with the four lag 0–3 being most consistently significant. We found a significant effect at lag 3, with a 0.9% increase (95% CI, 0.1–1.7) in total wheeze for 9.6 grains/m^3^ in Graminacee and a 2.6% increase (95% CI, 0.05–5.3) in gastroenteric disorders for 27.7 grains/m^3^ in Urticaceae.

The varying widths of CIs seen in Figure 1 are attributable mainly to the difference in distributional characteristics of the exposure variables—those with narrow CIs tend to be the exposure variables with right-skewed distributions, whereas those with wide confidence bands tend to be those with more normally distributed variables.

## Discussion

The present study shows a significant association between hospital emergency visits for wheezing and gastroenteric disorders in children 0–2 years of age and air pollution levels in six urban cities in Italy, located in different geographical areas (northern, central and southern Italy, plus seaside localities and hinterland territory) having different climatic conditions.

Very young children represent a population more susceptible to adverse health effects; the immune system in the early ages of life is still underdeveloped, as it must recognize the newly assimilated foods during the weaning period. Furthermore, children having a lesser corporeal surface but a higher respiratory frequency inhale and absorb more pollutants in relation to their weight compared with adults.

However, only a few studies have investigated the respiratory effects of air pollution among very young children to date. A recent study in Copenhagen (Andersen et al. 2008) found an association between incident wheezing symptoms in infants (0–1 years of age) and air pollution (PM_10_, NO_2_, CO) with 3- to 4-day lag, consistent with the delayed associations found in our study. Barnett et al. (2005) analyzed data on respiratory hospital admissions in children for three age groups (< 1, 1–4, 5–14 years) in five cities in Australia and two in New Zealand. They found significant association between air pollution (PM_2.5_, PM_10_, NO_2_, and SO_2_) and hospitalizations for pneumonia and acute bronchitis for the age groups < 1 and 1–4 years and all respiratory diseases for the three age groups. Pollution levels in those countries were lower than those observed in Italian cities. Villeneuve et al. (2007) examined associations between air pollution and ER visits for asthma among children (2–4, 5–14 years of age) and adults (e.g., 15–24, 25–44 years of age) and reported that the air pollution associations were strongest among young children, with NO_2_ and CO having especially pronounced associations. For the 2- to 4-year-old group, CO showed the strongest associations in the warm season, and the estimated risks increased as longer lags were included in the moving averages, which is also consistent with our finding.

More studies examined either older children or children as defined with wider age ranges. These include two studies from Italian cities. Vigotti and colleagues (2007) investigated associations between air pollution and ER visits for respiratory complaints for children (< 10 years of age) and the elderly (> 65 years of age) in Pisa and found significant increase in the ER visits and with increases in PM_10_ and NO_2_ (CO was positive but not significantly associated). Similarly, Bedeschi et al. (2007) found increases in ER visits for respiratory diseases among children < 15 years of age associated with elevated levels of PM_10_ and NO_2_, with a magnitude of excess risks comparable with those found in our study. In the study by Bedeschi et al., the associations appeared to increase or persist at longer lags (up to 5 days), which is also consistent with our finding. Thus, the results from the Italian studies that involved older children are consistent with the finding from our study with very young children.

In our study, CO showed the strongest associations with ER visits for wheezing, followed by SO_2_. However, PM_10_ and NO_2_ also showed consistently positive risk estimates (though not statistically significant) with the lag structure of associations similar to those for CO and SO_2_. In the studies that we mentioned above, as well as in other studies that found associations between air pollution and children’s respiratory morbidity—such as the analysis by Luginaah et al. (2005) in Windsor, Ontario, Canada, or the study by Lin et al. (2005) in Toronto, Ontario, Canada—researchers found associations with similar groups of air pollutants, generally including two or more from CO, NO_2_, SO_2_, and some PM indices. These pollutants likely share the same temporal fluctuations due to air stagnation, but they also represent local combustion sources including traffic. Thus, it may be more reasonable to consider these pollutants as surrogate indicator(s) of traffic and local combustions than to attempt to seek independent effects of single pollutants. It is also worth noting that the main source of pollution in five of these urban areas is traffic, whereas one of them is exposed also to industrial sources.

A study conducted by Brauer and co-investigators (2002) in the Netherlands, though different in the study design, found an association between residing near motorways with intense road traffic and a higher prevalence in respiratory infections with wheezing and asthma in children in the same age group as in our study (0–2 years of age). Studies are needed to investigate connections between short-term associations, incidence, and prevalence of these respiratory outcomes.

Another interesting result of our study is the association between air pollution and gastroenteric emergency visits. This is the first study to report this association in children. Previous studies (Chen et al. 2000; Lipsett et al. 1997), which examined ER visits for gastroenteritis as a control group, did not find association with the gaseous pollutants or PM indices. The mechanisms underlying these effects are not well known. Gastroenteritis is an inflammation of the gastrointestinal tract that could be caused by infection or by adverse reaction to ingested or inhaled material (Kasper et al. 2005). It is possible that particles are involved in the mechanism. Poorly soluble particles deposited in the oral passages may be cleared by coughing and expectoration or by swallowing into the gastrointestinal tract. Soluble particles are likely to be rapidly absorbed after deposition, but deposition depends on the rate of dissolution of the particle and the molecular size of the solute (U.S. Environmental Protection Agency 2004). Our study focused on very young children, who are still developing. Therefore, our findings might reflect the susceptibility of this age group. Clearly, more studied are needed to replicate our finding in this age group and to understand the possible mechanisms.

Public health implication of the impact of air pollution on wheezing at a very young age may be profound. Viral infections determining wheezing are frequent conditions in children < 3 years of age, and in case of an increased individual genetic susceptibility (Martinez 2005), an abnormal reaction may occur because of the immaturity of the immune response, thus facilitating the onset of chronic obstructive pulmonary disease (COPD) in adult ages. Further research is needed to investigate the mechanism(s) contributing to the interactions between viral infections and the exposure to ambient air pollution, to help prevent wheezing in children and the possible onset of COPD in adult ages.

The limitations of our study include relatively small counts of the outcomes studied, as reflected in the wide CIs—a tradeoff when investigating the outcomes at a very narrow age interval. Also, the particle indices available (TSP and PM_10_) are somewhat limited in that there were no data available on fine particles or their chemical constituents, which would have allowed a better characterization of the type of air pollution that may be responsible for the observed associations.

In conclusion, we found association between hospital emergency visits for wheezing and gastroenteric disorders in children 0–2 years of age and air pollution levels in urban cities in Italy. Local combustion sources, including traffic, may be responsible for the observed associations. Further research is needed to investigate the impact of air pollution on very young children, as they may also influence their health conditions at a later stage of life.

## Figures and Tables

**Table 1:** Descriptive statistics for daily counts of ER visits in each city, in total and by season (mean ± SD).

	Ancona	Bologna	Florence	Naples	Padua	Varese–Gallarate
Total wheeze						
All	0.7 ± 1.0	3.6 ± 3.4	1.9 ± 2.2	18.3 ± 9.1	4.8 ± 4.9	1.0 ± 1.3
Winter	0.9 ± 1.1	4.7 ± 3.7	2.6 ± 2.6	22.1 ± 10.1	6.6 ± 5.7	1.3 ± 1.4
Summer	0.6 ± 0.9	2.4 ± 2.5	1.1 ± 1.3	14.6 ± 5.8	3.0 ± 2.8	0.7 ± 1.0
No. of admissions	1,337	6,526	4,776	33,501	5,299	1,833
Total gastroenteric disorders						
All	0.4 ± 0.7	1.7 ± 1.6	0.9 ± 1.2	8.0 ± 3.8	2.9 ± 2.4	0.5 ± 0.8
Winter	0.3 ± 0.6	1.9 ± 1.8	1.1 ± 1.4	6.6 ± 3.0	2.9 ± 2.4	0.6 ± 0.9
Summer	0.4 ± 0.7	1.5 ± 1.4	0.8 ± 1.0	9.4 ± 4.0	2.9 ± 2.4	0.5 ± 0.7
No. of admissions	641	3,102	2,372	14,626	3,170	1,003
Years of study	1996–2000	1996–2000	1996–2002	1996–2000	1996–1998	1996–2000

**Table 2:** Mean ± SD for environmental variables in six cities.

	Ancona	Bologna	Florence	Naples	Padua	Varese–Gallarate
Temperature (°C)						
All	14.6 ± 7.1	16.2 ± 9.1	15.3 ± 7.2	18.6 ± 7.0	15.1 ± 7.4	12.7 ± 7.5
Winter	9.5 ± 4.4	9.7 ± 5.7	9.8 ± 4.7	13.9 ± 4.7	9.4 ± 4.4	6.5 ± 4.5
Summer	19.7 ± 5.4	22.6 ± 7.2	20.7 ± 4.8	23.3 ± 5.7	21.0 ± 4.8	18.4 ± 4.6

Relative humidity (%)						
All	60.8 ± 21.1	69.0 ± 12.0	72.6 ± 15.2	44.0 ± 22.9	83.2 ± 7.3	70.7 ± 18.6
Winter	63.4 ± 21.0	72.5 ± 12.5	76.9 ± 15.8	44.4 ± 23.6	84.6 ± 6.6	73.3 ± 19.6
Summer	58.2 ± 20.8	65.6 ± 10.6	68.1 ± 13.1	43.5 ± 22.3	81.9 ± 7.6	68.3 ± 17.4

PM_10_ (μg/m^3^)						
All	43.2 ± 42.1	50.8 ± 26.1	43.8 ± 18.9	44.5 ± 18.3	48.1 ± 13.5	63.2 ± 22.1
Winter	38.1 ± 27.4	61.7 ± 28.4	46.5 ± 20.0	39.4 ± 18.4	46.9 ± 14.6	67.6 ± 25.2
Summer	48.3 ± 52.1	40.1 ± 18.0	41.0 ± 17.2	49.6 ± 16.8	49.4 ± 12.0	59.1 ± 17.7

NO_2_ (μg/m^3^)						
All	42.5 ± 32.9	64.8 ± 20.3	57.9 ± 17.8	78.6 ± 30.6	48.7 ± 18.2	40.8 ± 17.0
Winter	47.5 ± 35.2	73.3 ± 19.2	63.5 ± 19.1	86.8 ± 35.6	55.2 ± 20.3	49.0 ± 18.1
Summer	37.5 ± 29.5	56.4 ± 17.8	52.3 ± 14.4	70.5 ± 21.9	42.1 ± 12.8	33.1 ± 11.4

SO_2_ (μg/m^3^)						
All	14.6 ± 9.8	7.2 ± 6.0	5.5 ± 4.3	21.1 ± 25.2	17.3 ± 7.3	7.0 ± 5.9
Winter	14.7 ± 10.8	10.1 ± 6.7	6.9 ± 5.2	18.5 ± 19.3	19.1 ± 8.2	10.9 ± 6.1
Summer	14.5 ± 8.6	4.4 ± 3.3	4.1 ± 2.5	23.6 ± 29.6	15.4 ± 5.6	3.5 ± 2.6

CO (μg/m^3^)						
All	2.1 ± 0.9	1.4 ± 0.9	1.5 ± 0.8	2.6 ± 1.3	1.9 ± 0.9	1.3 ± 0.8
Winter	2.3 ± 0.8	1.8 ± 1.0	1.9 ± 0.8	3.0 ± 1.6	2.4 ± 0.9	1.8 ± 0.8
Summer	1.9 ± 0.9	0.9 ± 0.5	1.1 ± 0.4	2.3 ± 1.0	1.4 ± 0.4	0.8 ± 0.3

O_3_ (μg/m^3^)						
Winter	30.7 ± 30.7	23.9 ± 40.5	22.5 ± 16.7	54.7 ± 18.5	34.6 ± 18.8	23.5 ± 17.9
Summer	41.3 ± 29.6	72.9 ± 40.5	60.7 ± 19.1	86.8 ± 32.4	55.9 ± 20.3	69.0 ± 26.9

Gramineae (grains/m^3^)						
Summer	2.6 ± 10.7	25.1 ± 39.2	4.8 ± 12.7	4.2 ± 9.1	4.4 ± 9.8	31.5 ± 113.1

Urticaceae (grains/m^3^)						
Summer	37.9 ± 44.4	36.9 ± 65.9	2.7 ± 6.8	59.9 ± 95.0	15.4 ± 32.5	12.1 ± 27.3

**Table 3:** Percentage increase (95% CI) in risk of total wheeze for an IQR increase in air pollution: combined results across six cities and *p*-value for homogeneity test.

Pollutant	Percent (95% CI)	IQR	*p*-Value for homogeneity
CO lag 0–1	1.7 (0.2 to 3.3)	1.1	0.85
CO lag 0–2	2.2 (0.5 to 3.9)	1.1	0.76
CO lag 0–3	2.3 (0.5 to 4.1)	1.1	0.50
CO lag 0–4	2.1 (0.2 to 4.0)	1.1	0.48
CO lag 0–5	2.4 (0.1 to 4.8)	1.0	0.37
CO lag 0–6	2.7 (0.5 to 4.9)	1.0	0.41

NO_2_ lag 0–1	1.4 (−1.6 to 4.4)	26.0	0.02
NO_2_ lag 0–2	2.1 (−1.3 to 5.7)	24.9	< 0.001
NO_2_ lag 0–3	2.3 (−1.4 to 6.2)	24.0	< 0.001
NO_2_ lag 0–4	2.7 (−1.1 to 6.6)	23.2	< 0.001
NO_2_ lag 0–5	2.6 (−1.2 to 6.7)	22.8	< 0.001
NO_2_ lag 0–6	2.8 (−1.0 to 6.7)	22.2	0.02

PM_10_ lag 0–1	1.8 (−2.0 to 5.7)	21.3	< 0.001
PM_10_ lag 0–2	1.7 (−2.9 to 6.4)	20.7	< 0.001
PM_10_ lag 0–3	2.5 (−2.6 to 7.8)	20.1	< 0.001
PM_10_ lag 0–4	2.9 (−2.9 to 9.0)	19.7	< 0.001
PM_10_ lag 0–5	3.4 (−2.5 to 9.8)	19.3	< 0.001
PM_10_ lag 0–6	3.8 (−2.3 to 10.3)	18.9	< 0.001

SO_2_ lag 0–1	0.1 (−1.4 to 1.6)	8.7	0.85
SO_2_ lag 0–2	0.9 (−0.7 to 2.5)	8.5	0.90
SO_2_ lag 0–3	1.7 (0.0 to 3.4)	8.3	0.82
SO_2_ lag 0–4	2.1 (0.4 to 3.9)	8.2	0.54
SO_2_ lag 0–5	2.8 (0.9 to 4.6)	8.1	0.52
SO_2_ lag 0–6	3.4 (1.5 to 5.3)	8.0	0.61

O_3_ lag 0–1	−1.9 (−6.6 to 3.1)	42.1	0.11
O_3_ lag 0–2	−3.1 (−8.9 to 3.1)	41.5	0.03
O_3_ lag 0–3	−2.9 (−9.5 to 4.1)	41.7	0.02
O_3_ lag 0–4	−3.7 (−11.2 to 4.5)	41.5	> 0.001
O_3_ lag 0–5	−4.4 (−13.3 to 5.5)	41.5	> 0.001
O_3_ lag 0–6	−4.6 (−15.2 to 7.4)	41.6	> 0.001

Summer only Gramineae	0.4 (−0.5 to 1.2)	9.6	0.52
Lag 1	0.4 (−0.4 to 1.2)	9.6	0.61
Lag 2	0.6 (−0.6 to 1.7)	9.6	0.22
Lag 3	0.9 (0.1 to 1.7)	9.6	0.45
Lag 0–1	0.5 (−0.5 to 1.6)	10.2	0.54
Lag 0–2	0.7 (−0.5 to 1.9)	10.4	0.45
Lag 0–3	1.2 (−0.1 to 2.5)	10.7	0.43

Urticaceae	0.3 (−0.3 to 1.0)	27.7	0.54
Lag 1	0.4 (−0.3 to 1.0)	27.7	0.44
Lag 2	0.0 (−0.6 to 0.7)	27.7	0.86
Lag 3	0.4 (−0.2 to 1.1)	27.7	0.70
Lag 0–1	0.5 (−0.3 to 1.3)	28.2	0.52
Lag 0–2	0.5 (−0.5 to 1.4)	29.9	0.53
Lag 0–3	0.7 (−0.4 to 1.8)	30.8	0.71

**Table 4:** Percentage increase (95% CI) in risk of gastroenteric disorders for an IQR increase in air pollution: combined results across the six cities, and *p*-value for homogeneity.

Pollutant	Percent (95% CI)	IQR	*p*-Value for homogeneity
CO lag 0–1	2.7 (0.1 to 5.4)	1.1	0.71
CO lag 0–2	3.8 (1.0 to 6.8)	1.1	0.85
CO lag 0–3	4.9 (−1.7 to 11.9)	1.1	0.27
CO lag 0–4	4.7 (−7.0 to 17.8)	1.1	0.05
CO lag 0–5	3.5 (−8.5 to 17.0)	1.0	0.05
CO lag 0–6	2.7 (−12.0 to 20.0)	1.0	0.01

NO_2_ lag 0–1	−1.1 (−3.2 to 1.1)	26.0	0.57
NO_2_ lag 0–2	0.1 (−3.0 to 3.3)	24.9	0.26
NO_2_ lag 0–3	1.8 (−2.4 to 6.2)	24.0	0.08
NO_2_ lag 0–4	2.9 (−1.6 to 7.6)	23.2	0.07
NO_2_ lag 0–5	2.3 (−1.9 to 6.6)	22.8	0.14
NO_2_ lag 0–6	2.5 (−1.7 to 6.9)	22.2	0.16

PM_10_ lag 0–1	2.4 (−1.0 to 5.8)	21.3	0.28
PM_10_ lag 0–2	2.4 (−1.4 to 6.3)	20.7	0.21
PM_10_ lag 0–3	2.7 (−1.5 to 7.0)	20.1	0.15
PM_10_ lag 0–4	2.9 (−1.9 to 7.9)	19.7	0.08
PM_10_ lag 0–5	3.2 (−1.6 to 8.3)	19.3	0.09
PM_10_ lag 0–6	3.8 (−1.6 to 9.4)	18.9	0.04

SO_2_ lag 0–1	−0.1 (−2.5 to 2.3)	8.7	0.78
SO_2_ lag 0–2	0.2 (−2.3 to 2.8)	8.5	0.84
SO_2_ lag 0–3	1.0 (−1.6 to 3.7)	8.3	0.50
SO_2_ lag 0–4	4.1 (−0.5 to 9.0)	8.2	0.16
SO_2_ lag 0–5	7.0 (0.1 to 14.3)	8.1	0.06
SO_2_ lag 0–6	8.5 (0.6 to 16.9)	8.0	0.04

O_3_ lag 0–1	2.1 (−3.8 to 8.4)	42.1	0.15
O_3_ lag 0–2	2.0 (−4.4 to 8.8)	41.5	0.16
O_3_ lag 0–3	4.0 (−3.9 to 12.5)	41.7	0.08
O_3_ lag 0–4	4.2 (−5.2 to 14.5)	41.5	0.03
O_3_ lag 0–5	5.6 (−4.5 to 16.7)	41.5	0.03
O_3_ lag 0–6	6.5 (−3.8 to 17.8)	41.6	0.06

Summer only Gramineae	−0.3 (−1.6 to 0.9)	9.6	0.98
Lag 1	0.4 (−0.8 to 1.6)	9.6	0.46
Lag 2	0.8 (−1.2 to 2.9)	9.6	0.05
Lag 3	1.0 (0.0 to 2.1)	9.6	0.66
Lag 0–1	0.1 (−1.5 to 1.6)	10.2	0.74
Lag 0–2	0.7 (−1.7 to 3.0)	10.4	0.18
Lag 0–3	1.2 (−1.2 to 3.7)	10.7	0.21

Urticaceae	0.0 (−1.8 to 1.9)	27.7	0.12
Lag 1	0.7 (−1.3 to 2.8)	27.7	0.07
Lag 2	0.0 (−1.0 to 1.0)	27.7	0.46
Lag 3	2.6 (0.0 to 5.3)	27.7	0.01
Lag 0–1	0.7 (−1.9 to 3.3)	28.2	0.05
Lag 0–2	0.7 (−1.9 to 3.5)	29.9	0.11
Lag 0–3	1.9 (−1.4 to 5.4	30.8	0.06
